# Nevus Unius Lateris: A Case Report

**DOI:** 10.7759/cureus.4481

**Published:** 2019-04-16

**Authors:** Kharel Narine, Litzel Carrera

**Affiliations:** 1 Preventive Medicine, Caja Del Seguro Social, Panama City, PAN; 2 Internal Medicine, Hospital Nacional, Panama City, PAN

**Keywords:** nevus unius lateris, epidermal verrucous nevus, dysembroplasia

## Abstract

Nevus unius lateris is a rare congenital hamartoma derived from the ectoderm, considered to be a systematized verrucous variant of an epidermal nevus. Due to its extensive unilateral distribution, it is frequently associated with neurological, musculoskeletal, auditory, and visual abnormalities. A case report of a 25-year-old female patient with a diagnosis of nevus unius lateris without associated comorbidities is presented.

## Introduction

Epidermal verrucous nevus is a common clinical finding; however, its generalized linear distribution known as nevus unius lateris is uncommon. There have been approximately 200 cases reported [1] worldwide and the pathogenesis still remains unknown. Clinically, lesions are characterized by confluent papillomatous, verrucous plaques distributed in a linear pattern that follow the Blaschko lines and are frequently associated with musculoskeletal, neurological, visual and auditory abnormalities, which manifest at birth or later during life [2]. Some cases have been reported where the patient does not develop symptoms other than those associated with traumatic injury to the lesions. Treatment is usually reserved for comorbidities due to the anti-aesthetic results obtained when trying to remove large nevi.

## Case presentation

**Figure 1:**
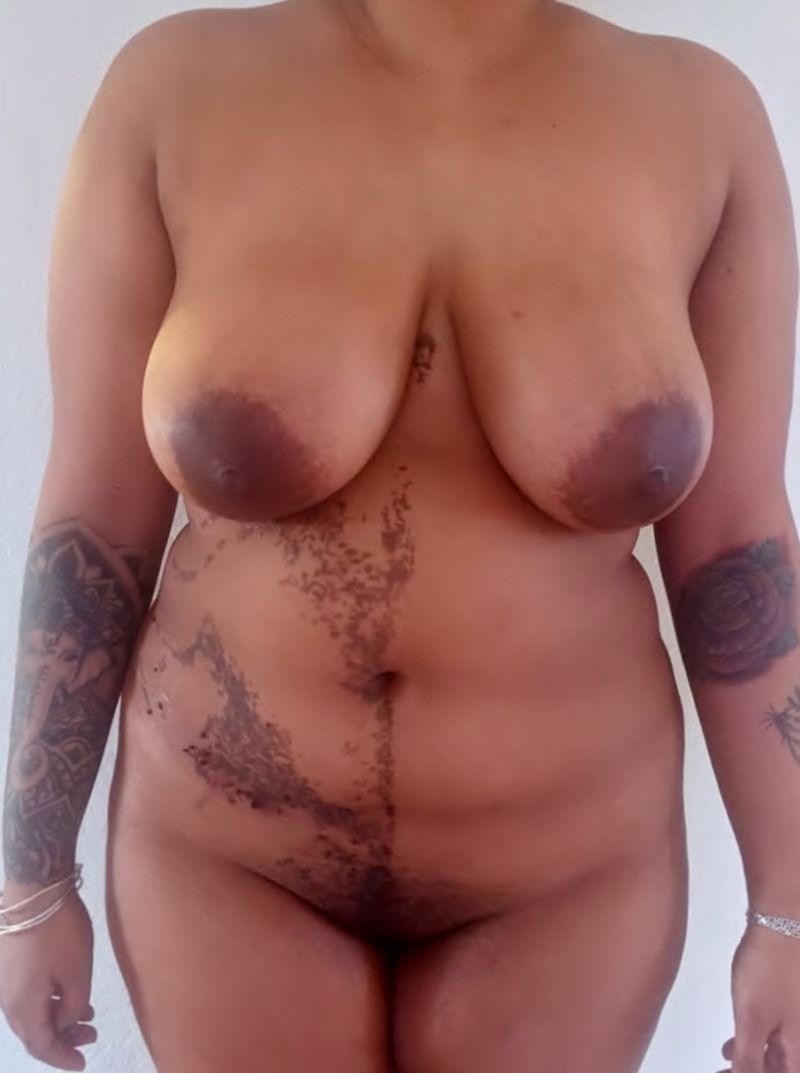
Hyperpigmented unilateral verrucous neoformations in the abdomen

**Figure 2:**
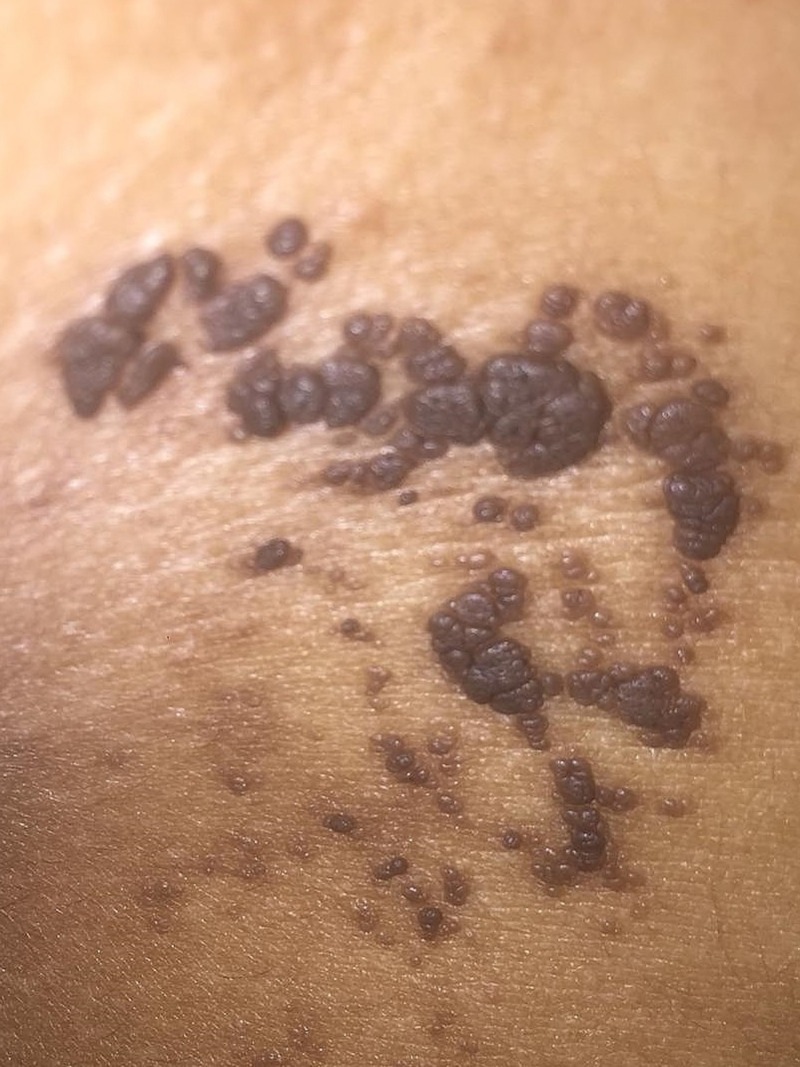
Close up of neoformations seen in previous figure

**Figure 3:**
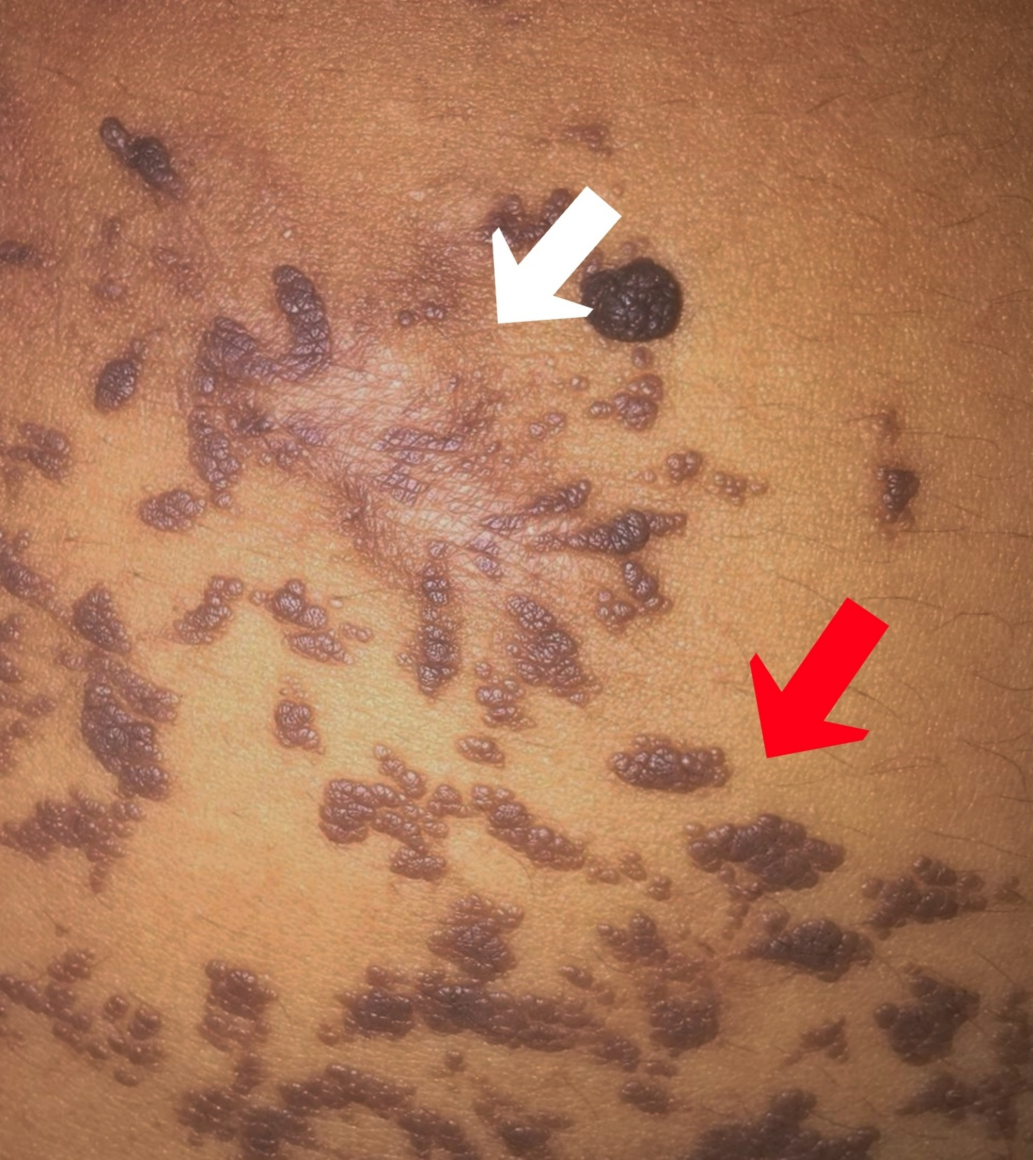
Results of treatment with electrofulguration and CO2 laser in a small area of the abdomen (white arrow) and untreated lesions for comparison (red arrow)

A 25-year-old female patient of Indian descent presented with verrucous hyperpigmented neoformations in the right hemibody at the level of the trunk, abdomen, back, genitals, groin, and leg, sparing the face, neck, and mucous membranes (Figures 1-2). The lesions described presented at birth and progressively increased in size and thickness. The patient's personal history was unremarkable, and maternal history was positive for a circumscribed epidermal verrucous nevus in the left forearm. After birth and subsequently during early infancy, routine blood and urine lab tests, neonatal and auditory screening tests, brain tomography scan without contrast and a skull X-ray were performed, all without pathologic findings. Psychomotor development was normal in all stages of life. The lesions remained asymptomatic during early childhood; however, as the lesions grew in size and became pedunculated, erosions and traumatic detachment occurred. At age 15, the patient received treatment with electrofulguration and CO2 laser treatments in a small area of the abdomen, with scarring and an unaesthetic appearance (Figure 3). The patient did not receive any more treatments due to the unwanted results and is asymptomatic to date. Physical examination at the time of presentation showed no abnormalities, other than the lesions previously described.

## Discussion

Epidermal verrucous nevus has an estimated prevalence in the general population of one in a thousand; however, its variant nevus unius lateris represents only 0.01 percent of this total [3]. Due to its extensive distribution, it is generally associated with neurological manifestations, musculoskeletal, auditory and visual disturbances (epidermal nevus syndrome or Solomon syndrome). Less commonly, this entity can manifest as an isolated finding, as portrayed in this report [4]. The etiopathogenesis of the disease still remains unknown; however, if the nevus follows Blaschko's lines it is considered mosaicism. A mutation has also been described in the FGFR3, HRAS or PIK3CA genes and a possible aberration in the long arm of chromosome one [5]. Familial cases are rare, [6]; there have not been previously documented evidence of heritability in similar case reports. The patient presented had a positive maternal history of similar lesions, and a benign course of the disease, making this the first reported case of an isolated nevus unius lateris with a familial component. The nevus unius lateris is characterized by small confluent hyperpigmented verrucous neoformations that cover a hemibody, always respecting the midline. Clinical diagnosis is the gold standard to date; nevertheless, in some cases, a biopsy may be required to confirm the diagnosis. A biopsy would report hyperkeratosis, papillomatosis, acanthosis, and lengthening of the interpapillary crests. The majority of the lesions remain asymptomatic; however, if they become pediculated and/or appear in flexor surfaces, there is a risk of traumatic detachment resulting in erosions and bacterial superinfection. The treatment of extensive lesions poses a great challenge. Surgical techniques, electrofulguration, cryotherapy, CO2 laser, photodynamic therapy, calcipotriol, and local and systemic retinoids can all be used. The results are variable and recurrences are frequent. In extensive cases, the results are anti-aesthetic. Verrucous lesions are replaced by scars, as in the case presented [7].

## Conclusions

Nevus unius lateris as an isolated dermatosis has rarely been described. It is a rare congenital dysembroplasia, which in most cases is associated with epilepsy, growth retardation, brain tumors and visual and auditory disturbances, making early diagnosis crucial. To the best of our knowledge, only two cases like this one have been previously reported, neither of which had a previous family history. Therefore, it is important to state that not all cases have a poor clinical prognosis and there can be a pattern of heritability, which had not previously been documented. Nonetheless, X-rays, computed tomography, and blood work should be done at birth and later in life to rule out associated disorders.
